# Understanding breast cancer patients' preference for two types of exercise training during chemotherapy in an unblinded randomized controlled trial

**DOI:** 10.1186/1479-5868-5-52

**Published:** 2008-10-27

**Authors:** Kerry S Courneya, Robert D Reid, Christine M Friedenreich, Karen Gelmon, Caroline Proulx, Jeffrey K Vallance, Donald C McKenzie, Roanne J Segal

**Affiliations:** 1 Faculty of Physical Education and Recreation, University of Alberta, Edmonton, Alberta, Canada; 2 Minto Prevention and Rehabilitation Center, University of Ottawa Heart Institute, Ottawa, Ontario, Canada; 3 Division of Population Health and Information, Alberta Cancer Board, Calgary, Alberta, Canada; 4 Department of Medical Oncology, British Columbia Cancer Agency, Vancouver, British Columbia, Canada; 5 Department of Medical Oncology, Ottawa Hospital Regional Cancer Center, Ottawa, Ontario, Canada; 6 Centre for Nursing and Health Studies, Athabasca University, Athabasca, Canada; 7 School of Human Kinetics, University of British Columbia, Vancouver, British Columbia, Canada

## Abstract

**Background:**

Patient preference for group assignment may affect outcomes in unblinded trials but few studies have attempted to understand such preferences. The purpose of the present study was to examine factors associated with breast cancer patients' preference for two types of exercise training during chemotherapy.

**Methods:**

Breast cancer patients (N = 242) completed a battery of tests including a questionnaire that assessed patient preference and the theory of planned behavior (TPB) prior to being randomized to usual care, resistance exercise training (RET), or aerobic exercise training (AET).

**Results:**

99 (40.9%) participants preferred RET, 88 (36.4%) preferred AET, and 55 (22.7%) reported no preference. Past exercisers (p = 0.023), smokers (p = 0.004), and aerobically fitter participants (p = 0.005) were more likely to prefer RET. As hypothesized, participants that preferred AET had more favorable TPB beliefs about AET whereas participants that preferred RET had more favorable TPB beliefs about RET. In multivariate modeling, patient preference for RET versus AET was explained (R^2 ^= .46; p < 0.001) by the difference in motivation for RET versus AET (β = .56; p < 0.001), smoking status (β = .13; p = 0.007), and aerobic fitness (β = .12; p = 0.018). Motivational difference between RET versus AET, in turn, was explained (R^2 ^= .48; p < 0.001) by differences in instrumental attitude (β = .27; p < 0.001), affective attitude (β = .25; p < 0.001), and perceived behavioral control (β = .24; p < 0.001).

**Conclusion:**

Breast cancer patients' preference for RET versus AET during chemotherapy was predicted largely by a difference in motivation for each type of exercise which, in turn, was based on differences in their beliefs about the anticipated benefits, enjoyment, and difficulty of performing each type of exercise during chemotherapy. These findings may help explain patient preference effects in unblinded behavioral trials.

**Trial Registration:**

ClinicalTrials.gov Identifier NCT00115713.

## 

A double-blind randomized controlled trial is the gold standard research design for testing health interventions because it controls for possible placebo effects[1]. A double-blind trial is one in which neither the participants nor the providers are aware of who is receiving which intervention. Such blinding is very difficult, if not impossible, for many behavioral interventions including exercise[2]. A recent recommendation for applying the Consolidated Standards for Reporting Trials (CONSORT) to behavioral trials acknowledged the challenge of unblinded behavioral trials but the primary recommendation was to promote the blinding of outcome assessors[2]. Although blinding outcome assessors is important, behavioral researchers should also consider incorporating patient preference measures into their unblinded trials[3].

Patient preference for group assignment is a potentially important but often ignored factor in unblinded trials[4]. Participants assigned to their preferred intervention may have higher expectations of benefit (i.e., a placebo effect) because they feel better matched to the intervention or because they feel better able to complete the intervention[4]. Conversely, participants assigned to their non-preferred intervention may suffer "resentful demoralization" or have a negative placebo-like response (i.e., expectation of less benefit) based on their perceived needs and/or ability[4]. Patient preference may have a major impact on intervention effectiveness in unblinded trials but few behavioral trials have examined this issue despite the fact that behavioral interventions are only rarely able to be blinded.

We recently reported one of the first exercise trials to examine patient preference effects. The **S**upervised **T**rial of **A**erobic versus **R**esistance **T**raining (START) compared the effects of aerobic exercise training (AET) and resistance exercise training (RET) to usual care (UC) in 242 breast cancer patients initiating adjuvant chemotherapy [5-10]. Prior to randomization, we assessed patient preference for group assignment, which is the recommended approach for examining patient preference effects in randomized trials[3]. As hypothesized, we found a significant interaction between patient preference and group assignment on our primary endpoint of quality of life[7]. Specifically, patients that preferred RET improved quality of life only when assigned to RET whereas patients with no preference improved quality of life only when assigned to AET. Patients that preferred AET did not respond differently to the interventions. These results suggest that the effects of exercise training on quality of life in the START trial were strongly influenced by patient preference prior to randomization. Understanding why patients hold such preferences may help to explain these important effects. Few studies, however, have attempted to understand patient preferences in unblinded trials[4].

The purpose of the present study was to identify the key factors associated with a patient preference for RET versus AET in the START trial. Given that a preference expresses a difference in the desirability of two or more alternatives and is most often represented by the concept of attitude[4], we selected Ajzen's [11] Theory of Planned Behavior (TPB) to guide our investigation. The TPB proposes that intention is the most important determinant of a behavior. Intention is conceptualized as having two components: a behavioral choice or goal (i.e., what one intends to do) and intention strength (i.e., how motivated one is to do it). In the present study, we conceptualized patient preference as the behavioral choice component (i.e., what one would intend to do if given the choice) and we assessed motivation as an indicator of the strength of the behavioral intention. Intention, in turn, is influenced by perceived behavioral control (i.e., perceived ease or difficulty of performing the behavior), attitude (i.e., a positive or negative evaluation of performing the behavior including instrumental and affective components), and subjective norm (i.e., perceived approval/support for performing the behavior). Based on the TPB, we hypothesized that motivation would be the strongest determinant of patient preference and, in turn, would be influenced by instrumental attitude (expected benefits), affective attitude (expected enjoyment), perceived behavioral control (anticipated difficulty), and subjective norm (anticipated support). We also explored the effects of demographic and medical variables (e.g., age, disease stage, treatments), behavioral/fitness indicators (e.g., past exercise, smoking, aerobic fitness), and patient-rated outcomes (e.g., quality of life, depression).

## Methods

### Setting and participants

The methods and main results of the START trial have been reported elsewhere [5-10] and are summarized briefly here. Participants were recruited in Edmonton, Ottawa, and Vancouver, Canada. Ethical approval was obtained from the cancer centers and written informed consent was obtained from participants. Eligibility criteria were women ≥ 18 years old with stage I–IIIA breast cancer initiating adjuvant chemotherapy. Women were excluded if they had incomplete axillary surgery, transabdominal rectus abdominus muscle reconstructive surgery, uncontrolled illnesses, or were not approved for participation by their oncologist.

### Design and procedures

The study was a prospective, three-armed, randomized controlled trial. Eligible participants were identified by their treating oncologist. Participants were informed that they would be randomly assigned to AET, RET, or UC in a 1:1:1 ratio and that neither they, nor us, had any control over group assignment. Participants completed all baseline measures prior to being randomized.

### Exercise training interventions

Details of the exercise training interventions have been reported elsewhere[5]. The exercise programs were described to participants in the consent form and by the research coordinators during recruitment. Participants were informed that if they were assigned to AET or RET, they would be asked to exercise 3 times per week at a supervised fitness center for the duration of their chemotherapy. They were also informed about the specific types of exercises they would be asked to do. Specifically, for AET, they were informed that they could choose among treadmill, cycle ergometer, or elliptical machines. For RET, they were informed that they would be asked to perform nine different upper and lower body exercises on machines. Participants were informed that if they were assigned to UC, they would be offered 1 month of supervised exercise after the postintervention assessments.

### Assessments of patient preference, theory of planned behavior, and covariates

At baseline (prior to randomization), participants completed a self-administered questionnaire that assessed patient preference, TPB constructs, and covariates. Patient preference was assessed by the single item: "Which exercise program would you prefer if you had the choice?" Participants were asked to place a checkmark beside one of three options: aerobic exercise, resistance exercise, or no preference. Patient preference was scored as a single variable coded as -1 = aerobic exercise, 0 = no preference, and +1 = resistance exercise.

TPB constructs were assessed in the same questionnaire prior to randomization by all participants and for both aerobic and resistance exercise based on standard guidelines and measures[12]. Motivation was measured by asking "How motivated are you to do the aerobic (or weight training) exercise program?" with response options ranging from 1 (slightly motivated) through 4 (moderately motivated) to 7 (extremely motivated). Attitude was measured using two items each for instrumental (useful-useless, harmful-beneficial) and affective (unenjoyable-enjoyable, unpleasant-pleasant) attitudes. The stem for the attitude items was "I think that doing aerobic (or weight training) exercise during my chemotherapy treatment will be..." with response options ranging from 1 (extremely "useless/harmful/unenjoyable/unpleasant") to 7 (extremely "useful/beneficial/enjoyable/pleasant"). The two items for each scale were averaged for the score. Alpha coefficients for the two items were .76 (AET instrumental), .86 (AET affective), .88 (RET instrumental), and .90 (RET affective). Perceived behavioral control was measured by two items: "If I wanted to, I could easily do the aerobic (or weight training) exercise program during my chemotherapy" (1 = strongly disagree to 7 = strongly agree) and "For me to do the aerobic (or weight training) exercise program during my chemotherapy treatment will be" (1 = extremely difficult to 7 = extremely easy). The two items were averaged for the score. Alpha coefficients were .63 (AET) and .74 (RET). Subjective norm was measured by the single item: "Most people who are important to me would support me doing aerobic (or weight training) exercise during my chemotherapy treatment" and rated on a 7 point scale from 1 (strongly disagree) to 7 (strongly agree). We then computed TPB difference scores by subtracting the AET beliefs from the RET beliefs so that a positive score indicated a more favorable view of RET whereas a negative score indicated a more favorable view of AET.

Demographic data were collected by self-report and consisted of age (years), marital status (0 = not married; 1 = married), education (six categories), annual family income (six categories), employment status (0 = not employed full-time; 1 = employed full-time), and location/center (0 = Ottawa; 1 = Edmonton; 2 = Vancouver). Medical data were collected from medical records and consisted of disease stage (I, IIa, IIb, and IIIa), type of surgery (0 = lumpectomy; 1 = mastectomy), and type of chemotherapy (0 = nontaxane; 1 = taxane). Behavioral variables were collected from self-report and consisted of smoking behavior (0 = nonsmoker; 1 = smoker) and recent exercise assessed by the Leisure Time Exercise Questionairre[13] and coded as meeting or not meeting public health guidelines based on achieving either 60 minutes of vigorous intensity exercise per week or 150 minutes of moderate plus vigorous intensity exercise per week[14].

Measures of physical fitness are reported elsewhere [5] and included peak oxygen consumption (VO_2 peak_), muscular strength, body mass index, fat mass, and lean body mass. Patient-rated outcomes consisted of cancer-specific QoL and fatigue assessed by the Functional Assessment of Cancer Therapy-Anemia (FACT-An) scale[15], the Rosenberg Self-Esteem Scale[16], the Center for Epidemiological Studies-Depression Scale[17], and the Spielberger State Anxiety Inventory[18], each of which has been described elsewhere [5-10].

### Statistical analyses

We compared participants across the three patient preference groups (prefer AET, prefer RET, and no preference) on their TPB beliefs about AET and RET using analyses of variance (ANOVA). Significant ANOVAs were followed by Tukey post hoc tests. We also compared participants within each patient preference group on their differences in TPB beliefs about RET versus AET using dependent t-tests. We analyzed the associations between categorical variables (e.g., demographic, medical) and patient preference using chi-square analyses. For the multivariate analyses, we used two different multiple regression models to explain patient preference. In the first model, we included all the TPB beliefs separately for AET and RET. In the second model, we included all the TPB difference scores for each belief. For both these models, all the TPB variables, and only covariates that were statistically significant in univariate analyses, were forced into the multivariate models. We conducted three regression models to explain motivation – one to explain AET motivation, one to explain RET motivation, and one two explain the difference in RET versus AET motivation. Once again, all TPB variables were forced into the models.

## Results

Details about trial recruitment and baseline characteristics of the sample are reported elsewhere[5]. Briefly, we recruited 242 of 736 (33%) eligible participants that ranged in age from 25–78 years (mean = 49 years); 21% were obese, 37% were postmenopausal, 61% had disease stage II, 59% received breast conservation surgery, 31% received a taxane-based chemotherapy, and 25% reported exercising since their diagnosis with breast cancer. Patient preference for group assignment was roughly split between RET (n = 99; 40.9%) and AET (n = 88; 36.4%) with 22.7% (n = 55) reporting no preference[7].

### Comparison of theory of planned behavior beliefs across patient preference groups

TPB beliefs are presented in Table 1 overall and by patient preference group. For the AET beliefs, differences by patient preference group emerged for motivation (p = 0.003) and perceived behavioral control (p = 0.010). Tukey post hoc tests showed that participants that preferred AET were significantly more motivated to do AET compared to participants that preferred RET (p = 0.002). TPB beliefs about RET varied by patient preference group for all variables including affective attitude (p < 0.001), instrumental attitude (p < 0.001), subjective norm (p < 0.001), perceived behavioral control (p < 0.001), and motivation (p < 0.001). Tukey post hoc tests showed that, in all cases, participants that preferred RET had significantly more positive beliefs about RET compared to participants that preferred AET (all p values < 0.002). Moreover, compared to participants with no preference, participants that preferred RET also had significantly more positive RET beliefs for perceived behavioral control (p = 0.010) and motivation (p = 0.040), and borderline significantly more positive RET beliefs for instrumental attitude (p = 0.081) and affective attitude (p = 0.077). Finally, compared to participants that preferred AET, participants with no preference had significantly more positive RET beliefs for instrumental attitude (p = 0.005), subjective norm (p < 0.001), and motivation (p < 0.001).

**Table 1:** Beliefs About Aerobic and Resistance Exercise Training During Breast Cancer Chemotherapy, Overall and by Patient Preference for Group Assignment.

Variable	Overall (N = 242) M (SD)	Preferred AET (n = 88) M (SD)	Preferred RET (n = 99) M (SD)	No Preference (n = 55) M (SD)	P value
Aerobic Exercise Beliefs					
Affective attitude	5.4 (1.0)	5.6 (0.9)	5.3 (1.1)	5.2 (1.1)	.126
Instrumental attitude	6.3 (0.7)	6.3 (0.6)	6.3 (0.7)	6.2 (0.8)	.901
Subjective norm	6.6 (0.8)	6.6 (0.7)	6.6 (0.9)	6.7 (0.9)	.618
Perceived behavior control	4.5 (1.2)	4.5 (1.1)	4.7 (1.2)	4.1 (1.2)	.010
Motivation	5.5 (1.4)	5.8 (1.3)	5.2 (1.4)	5.5 (1.2)	.003
Resistance Exercise Beliefs					
Affective attitude	5.2 (1.2)	4.8 (1.2)	5.6 (1.0)	5.2 (1.1)	<.001
Instrumental attitude	6.0 (1.0)	5.6 (1.1)	6.4 (0.7)	6.1 (0.9)	<.001
Subjective norm	6.4 (1.1)	6.1 (1.3)	6.6 (1.0)	6.6 (0.9)	<.001
Perceived behavior control	4.4 (1.4)	3.9 (1.4)	5.0 (1.3)	4.3 (1.2)	<.001
Motivation	5.4 (1.5)	4.5 (1.7)	6.1 (1.0)	5.5 (1.3)	<.001

RET = resistance exercise training; AET = aerobic exercise training.

### Comparisons of theory of planned behavior beliefs within patient preference groups

**Figure 1:**
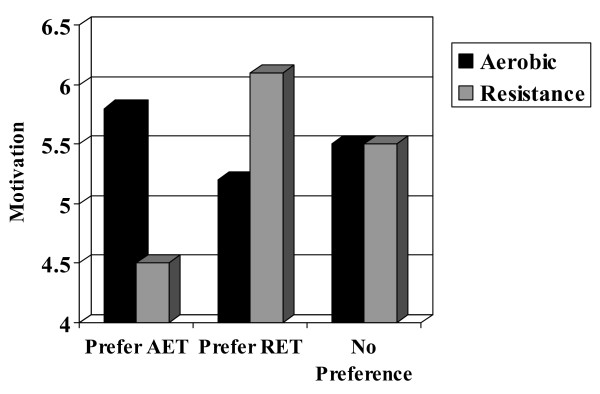
**Motivation for aerobic versus resistance exercise by patient preference.** Note: AET = aerobic exercise training; RET = resistance exercise training. Motivation assessed on a 7 point scale from 1 to 7.

Additional file 1 presents the within patient preference differences for each TPB belief. These analyses showed that participants that preferred AET had much more favorable beliefs about AET compared to RET for affective attitude (p < 0.001), instrumental attitude (p < 0.001), subjective norm (p < 0.001), perceived behavioral control (p < 0.001), and motivation (p < 0.001; Figure 1). Conversely, participants that preferred RET had significantly more favorable beliefs about RET compared to AET for affective attitude (p < 0.001), instrumental attitude (p = 0.027), perceived behavioral control (p = 0.022), and motivation (p < 0.001; Figure 1). Finally, participants with no preference showed minimal differences in their TPB beliefs about RET versus AET except a slightly more favorable instrumental attitude toward AET (p = 0.017).

### Associations between covariates and patient preference

**Figure 2:**
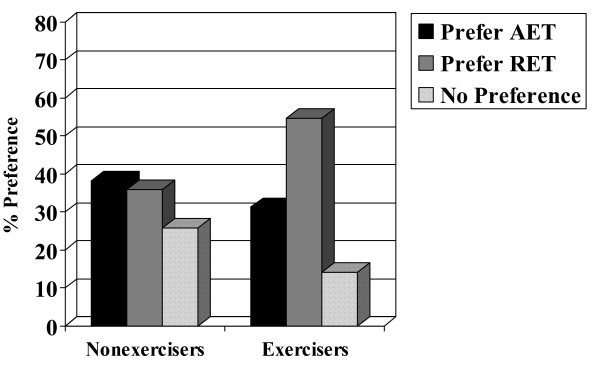
**Association between recent exercise and patient preference.** AET = aerobic exercise training; RET = resistance exercise training. Note: Exercisers defined as performing at least 150 minutes of moderate-to-vigorous exercise/week or 60 minutes of vigorous exercise/week.

**Figure 3:**
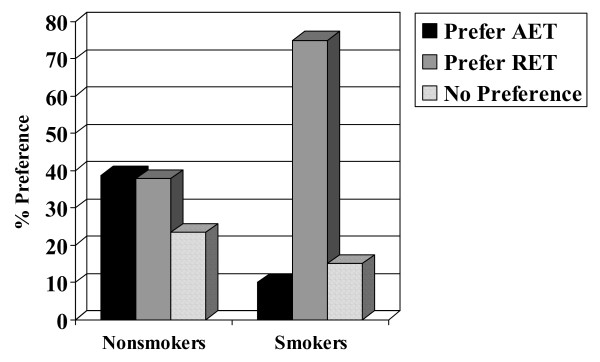
**Association between smoking and patient preference.** Note: AET = aerobic exercise training; RET = resistance exercise training.

**Figure 4:**
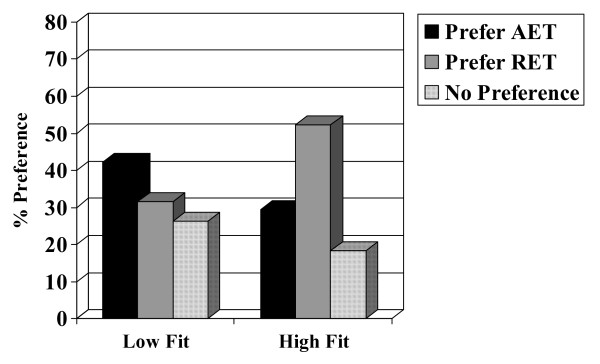
**Association between baseline aerobic fitness and patient preference.** Note: AET = aerobic exercise training; RET = resistance exercise training.

Chi-square analyses and analyses of variance indicated no associations with patient preference for age (p = 0.831), marital status (p = 0.483), education (p = 0.713), income (p = 0.540), employment status (p = 0.971), center/location (p = 0.524), disease stage (p = 0.970), type of surgery (p = 0.293), or chemotherapy protocol (p = 0.580). Moreover, there was no association with patient preference for muscular strength (p = 0.988), lean body mass (p = 0.831), fat mass (p = 0.945), or body mass index (p = 0.820). There were, however, associations with past exercise (p = 0.023), smoking (p = 0.004), and baseline aerobic fitness (p = 0.005). Specifically, past exercisers (Figure 2), smokers (Figure 3), and aerobically fitter participants (Figure 4) were more likely to prefer RET. No differences based on patient preference were found for self-esteem (p = 0.721), fatigue (p = 0.210), anxiety (p = 0.594), depression (p = 0.164), or quality of life (p = 0.097).

### Multivariate models of patient preference and motivation

Associations among the TPB beliefs ranged from small-to-large and were all statistically significant. For AET beliefs, the associations ranged from r = 0.14 (p = 0.028) for the subjective norm-affective attitude correlation, to r = 0.52 (p < 0.001) for the instrumental attitude-affective attitude correlation. For RET beliefs, the associations ranged from r = 0.28 (p < 0.001) for the subjective norm-affective attitude correlation, to r = 0.63 (p < 0.001) for the instrumental attitude-intention correlation. Finally, for the RET-AET belief differences, the associations ranged from r = 0.41 (p < 0.001) for the subjective norm-affective attitude correlation, to r = 0.64 (p < 0.001) for the instrumental attitude-affective attitude correlation. Univariate associations between each of the TPB beliefs and patient preference are presented in Table 2.

**Table 2:** Associations Among Exercise Beliefs and Patient Preference.

	Patient Preference^1^	AET Motivation	RET Motivation	RET-AET Motivation Difference^2^
				
	r (p value)	r (p value)	r (p value)	r (p value)
AET Beliefs				
Affective attitude	-.10 (p = .135)	.38 (p < .001)	.15 (p = .024)	-.20 (p = .002)
Instrumental attitude	.02 (p = .719)	.49 (p < .001)	.34 (p < .001)	-.09 (p = .152)
Subjective norm	-.02 (p = .755)	.30 (p < .001)	.14 (p = .030)	-.12 (p = .056)
Perceived control	.09 (p = .162)	.38 (p < .001)	.32 (p < .001)	-.02 (p = .776)
Motivation	-.22 (p = .001)	--	--	--
RET Beliefs				
Affective attitude	.29 (p < .001)	.21 (p = .001)	.49 (p < .001)	.30 (p < .001)
Instrumental attitude	.37 (p < .001)	.26 (p < .001)	.63 (p < .001)	.40 (p < .001)
Subjective norm	.21 (p = .001)	.14 (p = .035)	.39 (p < .001)	.27 (p < .001)
Perceived control	.34 (p < .001)	.17 (p = .010)	.54 (p < .001)	.39 (p < .001)
Motivation	.44 (p < .001)	--	--	--
RET-AET Belief Difference^2^				
Affective attitude	.47 (p < .001)	-.16 (p = .015)	.45 (p < .001)	.59 (p < .001)
Instrumental attitude	.45 (p < .001)	-.11 (p = .083)	.50 (p < .001)	.60 (p < .001)
Subjective norm	.28 (p < .001)	-.10 (p = .127)	.37 (p < .001)	.45 (p < .001)
Perceived control	.34 (p < .001)	-.22 (p = .001)	.35 (p < .001)	.54 (p < .001)
Motivation	.64 (p < .001)	--	--	--

^1^Patient preference coded as -1 (prefer AET), 0 (no preference), +1 (prefer RET). ^2^RET-AET difference calculated as RET belief minus AET belief. AET = aerobic exercise training; RET = resistance exercise training.

In the first multivariate model using separate AET and RET beliefs to explain patient preference, both AET and RET beliefs were forced into a multivariate regression analysis along with the three significant covariates (i.e., past exercise, smoking, aerobic fitness). The model explained 47% of the variance in patient preference with independent contributions from RET motivation (β = .52; p < 0.001), AET motivation (β = -.52; p < 0.001), smoking (β = .14; p = 0.005), and fitness (β = .11; p = 0.047). In the second model using the TPB belief differences to explain patient preference, 46% of the variance in patient preference was explained with independent contributions from motivation difference (β = .56; p < 0.001), smoking (β = .13; p = 0.007), and fitness (β = .12; p = 0.018).

Given the pre-eminence of motivation in understanding patient preference, we also conducted multivariate regression models for motivation. For AET motivation, the AET beliefs were forced into the model and explained 31% of the variance with the independent correlates being instrumental attitude (β = .31; p < 0.001), perceived behavioral control (β = .17; p = 0.006), subjective norm (β = .15; p = 0.011), and affective attitude (β = .14; p = 0.038). For RET motivation, the RET beliefs explained 48% of the variance with the independent correlates being instrumental attitude (β = .43; p < 0.001), perceived behavioral control (β = .26; p < 0.001), and subjective norm (β = .11; p = 0.049). Finally, for the motivation difference variable, the TPB difference beliefs explained 48% of the variance with the independent correlates being differences in instrumental attitude (β = .27; p < 0.001), affective attitude (β = .25; p < 0.001), and perceived behavioral control (β = .24; p < 0.001). Subjective norm was a borderline independent correlate in the model (β = .10; p = 0.072).

## Discussion

Our data show that breast cancer patients' preference for AET versus RET during chemotherapy was strongly associated with differences in their motivation for each type of exercise. Motivational differences, in turn, were largely based on differences in instrumental attitude (i.e., beliefs about how beneficial each type of exercise training would be during chemotherapy), affective attitude (beliefs about how enjoyable each type of exercise training would be during chemotherapy), perceived behavioral control (i.e., beliefs about how difficult each type of exercise training would be during chemotherapy), and, to a smaller extent, subjective norm (i.e., beliefs about how supportive others would be if they did each particular type of exercise training during chemotherapy). Consequently, our data suggest several possible explanations for a patient preference effect in unblinded behavioral trials.

First, there is the standard placebo effect. That is, participants assigned to their preferred exercise intervention may do better because they have a greater expectation of benefit than participants assigned to their non-preferred exercise intervention. Second, there is a possible "self-efficacy" effect wherein participants assigned to their preferred intervention may do better because they have a greater expectation of successfully completing the intervention than participants assigned to their non-preferred exercise intervention [4]. Third, there is a possible "enjoyment" effect based on anticipation of a more enjoyable experience for participants receiving their preferred intervention. Finally, there is a smaller "normative" effect wherein participants may do better in their preferred intervention because they expect more support from significant others if they were to do that intervention. The placebo effect is the standard explanation for unblinded drug trials whereas the self-efficacy, enjoyment, and normative effects would appear to be unique to behavioral trials in which the interventions themselves require significant effort, time, and skill on the part of participants, as well as support from significant others.

Interestingly, however, we previously noted that the patient preference effect reported in the START trial was only present for RET [7]. That is, patients that preferred RET only improved quality of life when assigned to RET but patients that preferred AET did not respond better to AET. We previously speculated that it is possible that a preference for RET is more stable because it is likely based on direct experience with AET whereas a preference for AET may be less stable because it is more likely based on a lack of experience with RET. Stable preferences are less likely to change over the course of an intervention [4]. The present study appears to provide some support for this hypothesis given that participants that were already exercising prior to the trial (which was almost exclusively aerobic exercise), or were more aerobically fit (demonstrating that they likely had previous experience with AET), were more likely to prefer RET.

These findings are also consistent with a "matching hypothesis". That is, RET preferers might have believed that RET would benefit them the most given their previous experience with AET. A "matching hypothesis" may also be supported by the association of smoking with RET preference. That is, it is possible that smokers preferred RET because they perceived that they were more able to perform RET and/or more likely to benefit from it. The matching hypothesis did not appear to be supported for participants that preferred AET although there was some evidence that those participants that were not exercising at baseline (i.e., no aerobic exercise) were more likely to express a preference for AET.

We also previously speculated that participants that preferred RET may have had a stronger preference than participants that preferred AET [7]. Stronger preferences are more likely to have substantive effects on outcomes[4]. Our data do not appear to support this hypothesis because the differences between AET and RET beliefs were much larger for participants that preferred AET than for participants that preferred RET (see Additional file 1). Theoretically, a preference is conceptualized as the difference in the desirability of two or more alternatives and, logically, the strength of the preference would be the degree of difference[4]. This larger differential within AET preferers, however, was based more on a negative evaluation of RET than a positive evaluation of AET. In fact, for absolute beliefs, RET preferers actually had more favorable views of RET than did AET preferers of AET. Participants that preferred RET simply did not have as negative a view of AET as did AET preferers of RET (see Table 1 and Figure 1). Consequently, it is unclear if a strong preference for an intervention is based on the absolute evaluation of an intervention or its relative evaluation compared to the alternative intervention. Our data suggest that the absolute evaluation of an intervention may be most important. That is, it may be more advantageous to receive an intervention that you really prefer – even if you only prefer it a little more than the alternative – than to receive an intervention that you only moderately prefer, even if you prefer it a whole lot more than the alternative.

We also previously reported that participants with no preference improved quality of life when assigned to AET compared to RET [7]. In the present study, participants with no preference had almost identical scores on beliefs about AET and RET, further supporting the true clinical equipoise of this group. Restricting randomization to only participants with a true clinical equipoise (i.e., no preference) has been suggested by some researchers[19] but this approach would have resulted in the exclusion of almost 80% of the participants in our trial. The alternative approach of assessing patient preference and examining its effects on outcomes as well as its determinants is not only more feasible in behavioral trials, but also more interesting, and more in line with the realities of clinical practice.

The strengths of our study include being the first study to prospectively examine patient preference for different types of exercise training during breast cancer chemotherapy, the adoption of a validated theoretical model to understand patient preference, and assessments of beliefs about both types of exercise training to allow a direct comparison of beliefs. The limitations of our study include the failure to obtain a measure of the strength of patient preference or change in patient preference over time, especially given that patients were undergoing chemotherapy. Moreover, although our model explained 46% of the variance in patient preference, there are clearly other important factors influencing patient preference that were not assessed in the present study. Another important limitation is that we did not include a patient preference option for UC. It is possible that some of our participants would have actually preferred UC although our clinical observation is that this number would have been small. This limitation may also have confounded our no preference option. That is, we assumed that participants that selected no preference were indicating that they had no preference between AET and RET, not that they had no preference between AET/RET and UC. Future unblinded behavioral trials should include an option for preferring UC even if UC is a no-treatment arm. Finally, our trial is limited in generalizability by the 33% recruitment rate and the focus on breast cancer patients. It is possible that patient preferences for exercise, and the determinants of such preferences, may vary based on many different demographic, medical, and psychosocial variables.

In summary, we examined factors associated with breast cancer patients' preferences for two types of exercise training during chemotherapy in an unblinded trial in which patient preference effects were observed[7]. We found that the majority of participants did, in fact, have a preference prior to randomization with a roughly equal split between AET and RET. Patient preference was strongly associated with the motivational difference between the two exercise interventions which, in turn, was based on differences in beliefs about the expected benefits, enjoyment, difficulty, and support for performing each type of exercise during chemotherapy. Our data provide evidence that patient preference effects in our trial may have resulted from a combination of placebo, self-efficacy, enjoyment, and normative effects, and that the absolute evaluation of an intervention may be more important than its relative evaluation compared to an alternative. Our study also supports the utility of the TPB as a conceptual model for understanding patient preference effects. Consequently, the TPB might also be usefully applied to other important intervention comparisons in exercise trials with the potential for patient preference effects such as home-based versus facility-based, unsupervised versus supervised, group versus individual, moderate versus vigorous intensity, and internet-delivered versus telephone-delivered. Examining, reporting, and understanding patient preference effects should be an integral part of unblinded behavioral trials.

## Competing interests

The authors declare that they have no competing interests.

## Authors' contributions

Professor Courneya had full access to all of the data in the study and takes responsibility for the integrity of the data and the accuracy of the data analysis. Study concept and design were undertaken by KSC, RDR, CMF, KG, DCM and RJS. Analysis and interpretation of data were undertaken by KSC, RDR, CMF, KG, CP, JKV, DCM and RJS. Drafting of the manuscript was undertaken by KSC. Critical revision of the manuscript for important intellectual content was undetaken by KSC, RDR, CMF, KG, CP, JKV, DCM and RJS. Statistical analysis was undertaken by KSC. Obtaining funding was undertaken by KSC, RDR, CMF, KG, DCM and RJS. Administrative, technical, or material support was undertaken by KSC, KG, DCM and RJS.

## Financial disclosures

None reported.

## Funding/support

The research for this article was funded by a grant from the Canadian Breast Cancer Research Alliance.

## Role of the funding source

The Canadian Breast Cancer Research Alliance had no role in the design and conduct of the study; collection, management, analysis, and interpretation of the data; and preparation, review, or approval of the manuscript.

## Supplementary Material

Additional file 1 **Differences in beliefs about aerobic and resistance exercise training during breast cancer chemotherapy.** Click here for file
