# Supplementary material for: Understanding breast cancer patients' preference for two types of exercise training during chemotherapy in an unblinded randomized controlled trial
Source: Int J Behav Nutr Phys Act. 2008 Oct 27;5:52. doi: 10.1186/1479-5868-5-52 (PMC2582030; doi:10.1186/1479-5868-5-52)
Supplement: Additional file 1 — Differences in beliefs about aerobic and resistance exercise training during breast cancer chemotherapy. [file 1479-5868-5-52-S1.doc]

Additional File 1.

Differences in Beliefs About Aerobic and Resistance Exercise Training During Breast Cancer Chemotherapy, Overall and by Patient Preference for Group Assignment.

--------------------------------------------------------------------------------------------------------------------------------------------------------------------------------------------------

Affective Instrumental Subjective Perceived

Attitude Attitude Norm Control Motivation

Difference Difference Difference Difference Difference

M [95% CI]; p value M [95% CI]; p value M [95% CI]; p value M [95% CI]; p value M [95% CI]; p value

--------------------------------------------------------------------------------------------------------------------------------------------------------------------------------------------------

Overall (N=242) -0.1 [-0.0 to- 0.3]; =.015 -0.2 [-0.1 to -0.3]; <.001 -0.2 [-0.1 to -0.3]; =.001 -0.1 [0.1 to -0.2]; =.237 -0.1 [0.1 to -0.3]; =.517

Preferred AET (n=88) -0.7 [-0.5 to -0.9]; <.001 -0.7 [-0.5 to -0.8]; <.001 -0.5 [-0.3 to -0.8]; <.001 -0.6 [-0.4 to -0.8]; <.001 -1.3 [-1.0 to -1.6]; <.001

Preferred RET (n=99) 0.3 [0.1 to 0.4]; <.001 0.1 [0.0 to 0.2]; =.027 0.0 [-0.1 to 0.2]; =.665 0.2 [0.0 to 0.4]; =.022 0.9 [0.7 to 1.2]; <.001

No Preference (n=55) -0.0 [0.2 to -0.2]; =.846 -0.2 [-0.0 to -0.3]; =.017 -0.1 [0.0 to -0.2]; =.067 0.2 [0.4 to -0.1]; =.120 0.1 [0.4 to -0.2]; =.604

--------------------------------------------------------------------------------------------------------------------------------------------------------------------------------------------------

Data are presented as the mean difference [95% confidence interval]. Difference is based on RET belief minus AET belief. AET=aerobic exercise training; RET=resistance exercise training; CI=confidence interval.
